# Brain‐derived neurotrophic factor attenuates cognitive impairment and motor deficits in a mouse model of Parkinson's disease

**DOI:** 10.1002/brb3.2251

**Published:** 2021-06-16

**Authors:** E Chang, Jiongmei Wang

**Affiliations:** ^1^ Department of Rehabilitation Medicine Cangzhou Central Hospital Cangzhou Hebei China

**Keywords:** BDNF, behavior, dopaminergic neuron, mitochondria, Parkinson's disease

## Abstract

**Background:**

Parkinson's disease (PD) is one of the most common neurodegenerative disorders that seriously impair the life quality and survival of patients. Herein, we aim to investigate the neuroprotective roles of brain‐derived neurotrophic factor (BDNF) in PD mice and reveal the underlying mechanisms. BDNF overexpression was achieved via the injection of adeno‐associated viruses (AAV) with BDNF gene.

**Methods:**

PD mouse model was established by 1‐methyl‐4‐phenyl‐1,2,3,6‐tetrahydropyridine (MPTP) treatment. Tests of rotarod, pole, open field, and novel object recognition were conducted to evaluate the motor and cognitive functions of treated mice.

**Results:**

Mitochondrial impairment, mitochondrial respiratory chain enzymes, and tyrosine hydroxylase (TH)‐positive dopaminergic neurons were detected to uncover the molecular mechanism. BDNF overexpression attenuated motor deficits and cognitive impairment in MPTP‐induced PD mice. Mechanistically, BDNF mitigated mitochondrial impairment increased the activity of respiratory chain Complex I and Ⅱ+III, and finally alleviated TH‐positive dopaminergic neuron loss in MPTP‐induced PD mice.

**Conclusion:**

This study highlights the potential of BDNF as a therapeutic candidate for the treatment of mitochondrial impairment‐associated neurodegenerative diseases.

## Graphical abstract

1. BDNF overexpression attenuated motor deficits and cognitive impairment in MPTP‐induced PD mice.
2. BDNF mitigated mitochondrial impairment, increased the activity of respiratory chain Complex I and II+III.

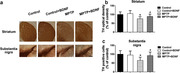

## 1 INTRODUCTION

Parkinson's disease (PD) is the second most common neurodegenerative disorder which affected approximately 7,000,000 people and caused 211,000 deaths globally in 2016 (GBD 2016 Neurology Collaborators, 2019; H. Li et al., 2020). As a progressive nervous system disorder, PD leads to motor and non‐motor symptoms, and the cases of PD are more common in the elderly. The rate in people over 60 years old is about 1% that increases to 4% of people over 80 years old (de Lau & Breteler, 2006). Motor symptoms include tremor, rigidity, slowness of movement, and freezing; behavioral problems, cognitive dysfunctions and memory loss are the most prevalent non‐motor characteristics of PD, which usually emerge slowly, and as the PD worsen, they become more common (Kalia, 2018; Sveinbjornsdottir, 2016). PD contributes to the global burden of neurodegenerative diseases and seriously impairs the life quality and survival of patients.

The main pathological characteristics of PD are death of dopaminergic neurons in the substantia nigra and the presence of Lewy bodies (Obeso et al., 2010; Prakash et al., 2016). The loss of neurons, with 70% of them being dopaminergic neurons, is accompanied by the cell death of microglia and astrocytes in the substantia nigra (Chen et al., 2019). Although the pathogenesis of PD is still not clear, cumulative evidence suggested that mitochondrial dysfunction played prominent role in the development of PD (Langston et al., 1983). Respiratory chain impairment is one of the major features in PD patients and proteins encoded by PD‐associated genes could affect the function of mitochondria. The 1‐methyl‐4‐phenyl‐1,2,3,6‐tetrahydropyridine (MPTP) is the mitochondrial toxin, which was found to cause PD like symptoms in patients (Langston et al., 1983). Following studies revealed that the active toxic metabolite of MPTP (MPP+) interfered with the activity of respiratory chain Complex I in dopaminergic neurons, and then resulted in neurodegeneration in substantia nigra of human and mouse (Langston et al., 1983; Schapira et al., 1989). Sixty percent of PD patients present Complex I deficiency and other components of the respiratory chain are also affected with 65% of PD patients displaying Complex II deficiency and 25–30% showing Complex IV impairment (Bury et al., 2017; Grunewald et al., 2016; Kraytsberg et al., 2006). These findings initiated the worldwide studies to explore the contribution of mitochondria to the pathology of PD. Multiple mitochondrial homeostasis‐related genes were found to strongly link the progression of PD, such as protein deglycase DJ‐1, PTEN‐induced putative kinase (PINK1), E3 ubiquitin ligase Parkin, and Leucine‐rich repeat kinase (LRRK2) (Larsen et al., 2018).

Numerous models have been created to study the pathogenesis of PD, and mouse models using MPTP are among the most widely used, which have shed light on the pathophysiology and behavioral deficits of PD and provided the model platforms for researchers to test symptomatic and neuroprotective drugs (Duty & Jenner, 2011; Meredith & Rademacher, 2011). BDNF is the most distributed neurotrophic factor in central nervous system which plays the key role in synaptic plasticity and neuron survival (Binder & Scharfman, 2004). Recently, the promising functions of BDNF in Alzheimer's disease (AD) have been reported (Giuffrida et al., 2018); however, the role of BDNF in PD is still unclear. In this study, we aim to demonstrate the protective function of BDNF on motor and cognitive functions in PD mouse model and reveal the underlying molecular mechanisms.

## 2 MATERIALS AND METHODS

### 2.1 Animals

Eight‐week‐old wild‐type (WT) mice on C57BL/6 background were ordered from Shanghai Model Organisms (Shanghai, China). Mice were housed under the 12/12 h light/dark cycles with free access of drinking water and chow diet. All animal studies were approved by the ethics committee of Cangzhou Central Hospital.

### 2.2 Adeno‐associated viruses (AAV)

The overexpression of BDNF in brain was carried out by using AAV‐BDNF, and AAV‐GFP was used as control. Briefly, target genes were sub‐cloned into pandora AAV‐9 shuttle vector and verified by DNA sequence. AAV‐BDNF and AAV‐GFP virus were generated and purified by OBiO Technology Corp., Ltd. (Shanghai, China). AAV titers were measured by using qRT‐PCR and simply blue safe staining was used to determine the purity of virus as previously described (Ayuso et al., 2010).

### 2.3 Brain stereotaxic injection

AAV virus was diluted to 0.6 × 10^9^ vg/μl. Each mouse was stereotaxically injected 5 μl BDNF‐AAV or GFP‐AAV into the left lateral ventricle with the stereotaxic coordinates (anteroposterior: −0.5 mm; lateral: 1.1 mm; ventral: 2.1 mm) by using the digital dual lab standard with mouse and neonatal rat adaptor (Stoelting Co., Wood Dale, USA).

### 2.4 Parkinson's disease model

**FIGURE 1:**
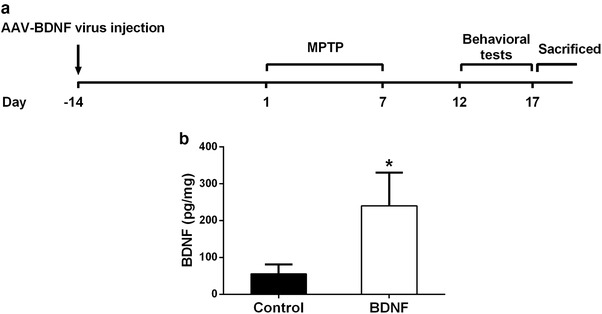
Establishment of BDNF overexpression. (a) Timeline of the experimental design. (b) Verification of viral‐mediated BDNF overexpression in mice brain by ELISA 2 weeks post AAV injection. Data were expressed as mean ± SD. *n* = 6 for each group. *P < .05 compared to control group

The mouse PD model was created by intraperitoneal injection of MPTP hydrochloride (#M0896, Sigma‐Aldrich, St. Louis, USA). Mice were treated with vehicle or MPTP (25 mg/kg) daily for 7 days. The above mice were divided into 4 groups randomly (*n* = 12 each group): control (GFP‐AAV + vehicle), control + BDNF (BDNF‐AAV + vehicle), MPTP (GFP‐AAV + MPTP), and MPTP + BDNF (BDNF‐AAV + MPTP). The timeline of the treatment is shown in Figure 1a.

### 2.5 Behavioral assessment

Behavioral assessment including rotarod performance test, pole test, open field test, and novel object recognition test were performed 5 days after the vehicle or MPTP treatment of the above groups of mice. Mice were habituated and trained 3 times a day for 5 days to acclimatize to the environments and tests. For rotarod performance test, YLC‐4C rotarod equipment was used to detect the motor coordination and degree of hypokinesia. Mice were placed on the rod for over 180 s which accelerated from 5 to 20 rpm. The stay on time (s) of each mouse was recorded as the latency period to fall, and mice were test 3 times with 10 min rest intervals. Pole test was performed to determine the ability to balance movement and the degree of bradykinesia of the treated mice. A 1‐cm diameter and 55‐cm height rough wooden pole was set upright and fixed. Mouse was positioned head upward near the top of the wooden pole, and the time when the mouse turned around and climbed down the pole was recorded. Each mouse was tested 3 times with 10 min rest intervals. To detect the exploratory activity and locomotor ability the open field test was performed as previously described (Y. Li et al., 2017). In brief, a 50‐cm × 50‐cm × 40‐cm square arena was separated into 25 equal‐sized squares with grids. Mouse was placed in the arena and was allowed to move freely. Within 5 min, the number of crossing line and rearing were recorded. Each mouse was tested 3 times with 10 min rest intervals. To investigate the ability of learning and memory in the treated mice, novel object recognition test was conducted as previously described (Lueptow, 2017). Briefly, two objects with same material were placed into box in a symmetrical position, and mouse was placed into this box for 1 h. Then one object was replaced with a novel object and the following behaviors were recorded: rearing on the object, sniffing the object (≤2 cm), and touching the object. Each mouse was tested 3 times with 10 min rest intervals. The recognition index was calculated as described before (Lueptow, 2017).

### 2.6 Immunoblotting analysis

Animals were euthanized at the end of behavioral test, and the whole brain of half of mice per group were collected immediately. The midbrain and striatum tissues were quickly dissected on ice. Total proteins were homogenized and lysed by using Beyotime RIPA buffer (Shanghai, China) supplemented with protease inhibitor cocktails. Expression levels of target proteins were determined by Western blot as described previously (Zhang et al., 2018). The primary antibody of Pink1 (ab23707, 1:1500 dilution) and Parkin (ab77924, 1:1000 dilution) were obtained from Abcam (Cambridge, UK). The β‐actin (#4970, 1:2000 dilution) primary antibody was purchased from Cell Signaling Technology, Inc. (Danvers, USA).

### 2.7 ELISA

Two weeks after the AAV injection, five mice of each group (control and BDNF) were used to detect the BDNF protein level in brain by using the Mouse BDNF ELISA Kit (MBS355435, MyBioSource, San Diego, USA) according to the manufacturer's instructions.

### 2.8 Activity of mitochondrial respiratory chain

The activity of mitochondrial respiratory chain Complex I, Complex II+III, and Complex IV were measured by using Cary‐1E spectrophotometer at 420, 600, and 550 nm respectively, following the methods described previously (Manfredini et al., 2019).

### 2.9 Immunohistochemistry analysis

Immunohistochemistry was conducted as previously described (Guo et al., 2019). In brief, the brain paraffin sections (30 μm) were incubated with Xylene and gradient Ethanol for deparaffination, and then incubated with H_2_O_2_ (3%) for 30 min. After the incubation, sections were blocked with blocking buffer (5% NGS and 3% BSA) for 2 h. Brain sections were incubated overnight with tyrosine hydroxylase (TH) primary antibody (ab112, 1:150 dilution) at 4°C, and then incubated by second antibody for 2 h and HRP‐streptavidin for 20 min at room temperature. Finally, brain sections were developed by DAB in 5–25 min and stained with hematoxylin and imaged by the inverted Olympus microscope IX71.

### 2.10 Statistical analysis

Statistical analyses were performed by using the GraphPad Prism 7.0 software package. One‐way analysis of variance (ANOVA) method with a Tukey's post hoc test was used to analyze the differences between groups. Each experiment was repeated at least three times and the data were represented as mean ± standard deviation (SD). *P < .05 compared to control group, ^#^ *P* < .05 compared to MPTP group.

## 3 RESULTS

### 3.1 BDNF attenuates motor deficits in MPTP‐induced PD mice

To investigate the protective role of BDNF in a mouse model with PD, recombinant GFP or BDNF gene was delivered into WT mice through AAV vector. Two weeks later, mice were intraperitoneally injected MPTP for 1 week to create the PD model. Five days after MPTP induction, mice were set for a series of behavioral tests, and then sacrificed for molecular analyses (Figure 1a). First, the BDNF protein level in brain was determined using ELISA , in comparison with control mice. BDNF protein was significantly increased in brain after stereotaxic injection of BDNF‐AAV (Figure 1b).

**FIGURE 2:**
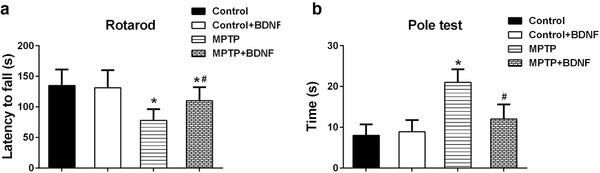
BDNF attenuated motor deficits in MPTP‐induced PD mice. (a) Latency to fall off the rotated rod. (b) Time to descend a pole. Data were expressed as mean ± SD. *n* = 12 for each group. *P < .05 compared to control group, ^#^ *P* < .05 compared to MPTP group

Both rotarod test and pole test were conducted to assess the motor deficits in MPTP‐induced PD mice. After 7 days of MPTP treatment, the time of latency to fall of MPTP mice was significantly reduced compared to that of Control mice (Figure 2a). Moreover, MPTP mice spent nearly twofold of time to complete the pole test (Figure 2b). Interestingly, overexpression BDNF in MPTP treatment mice could significantly increase the time of latency to fall and decrease the time of pole test (Figure 2a,b). These data suggested that overexpression BDNF in brain reduced cognitive impairment in MPTP‐induced PD mice.

### 3.2 BDNF reduces cognitive impairment in MPTP‐induced PD mice

**FIGURE 3:**
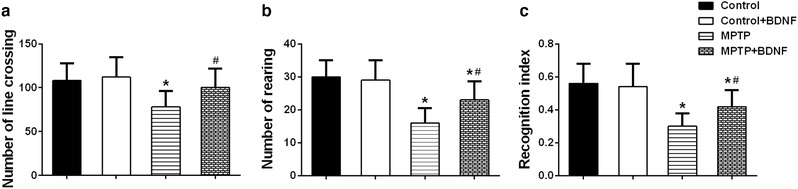
BDNF attenuated cognitive impairment in MPTP‐induced PD mice. (a) Number of line crossing in the open field test. Number of rearing (b) and recognition index (c) in the novel object recognition test. Data were expressed as mean ± SD. *n* = 12 for each group. *P < .05 compared to control group, ^#^ *P* < .05 compared to MPTP group

Next, we examined the cognitive ability of MPTP treated mice through open field and novel object recognition test. The number line crossing and rearing of MPTP treated mice were significantly less than those in control mice (Figure 3a,b). However, overexpression of BDNF in brain was able to increase the number of both line crossing and rearing in MPTP treated mice (Figure 3a,b). Additionally, novel object recognition test showed that the recognition index of MPTP treated mice was lower than that of mice in control group; similarly, BDNF overexpression significantly improved the ability of novel object recognition (Figure 3c). The above data indicated that overexpression of BDNF could reduce cognitive impairment in MPTP‐induced PD mice.

### 3.3 BDNF mitigates mitochondrial impairment in MPTP‐induced PD mice

**FIGURE 4:**
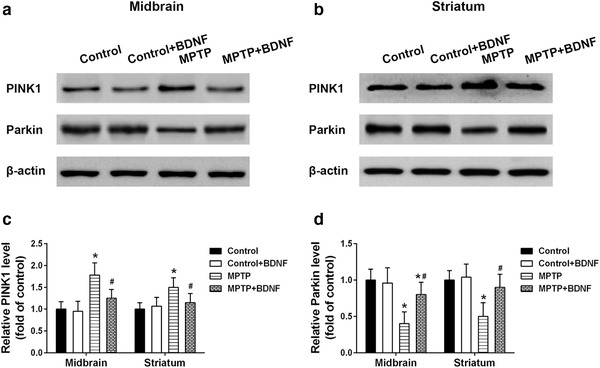
BDNF mitigated mitochondrial impairment in MPTP‐induced PD mice. Protein expression of PINK1 and Parkin in the midbrain (a) and striatum (b) of indicated mice. Relative expression levels of PINK1 (c) and Parkin (d) from Western blot were analyzed. Data were expressed as mean ± SD. *n* = 6 for each group. *P < .05 compared to control group, ^#^ *P* < .05 compared to MPTP group

Since mitochondrial impairment is one of major characteristic of PD, we investigated the fidelity of mitochondria in the treated mice by Western blot analysis. PINK1 usually accumulate on the outer membrane of damaged mitochondria and activate/recruit Parkin, an important protein that govern mitochondrial quality control, to the dysfunctional mitochondria (Pickrell & Youle, 2015). Western blot results showed that PINK1 was increased in midbrain and striatum after 7 days of MPTP treatment; however, overexpression BDNF could significantly reduce the expression of PINK1 (Figure 4a–c). In contrast, MPTP treatment decreased the protein levels of Parkin in both midbrain and striatum; however, BDNF overexpression was able to rescue the MPTP‐induced reduction of Parkin protein level (Figure 4a,b,d).

**FIGURE 5:**
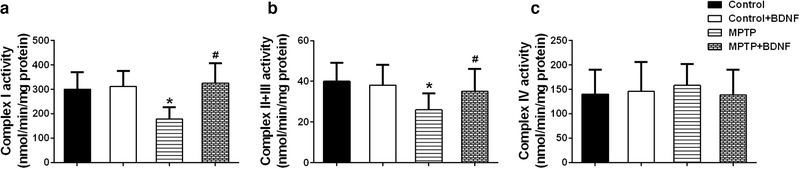
Effects of BDNF on mitochondrial respiratory chain enzyme activities. Activities of Complex I (a), Complex Ⅱ+III (b), and Complex IV (c) in the striatum were analyzed. Data were expressed as mean ± SD. *n* = 6 for each group. *P < .05 compared to control group, ^#^ *P* < .05 compared to MPTP group

Next, we examined the function of mitochondria in the treated mice by measuring the activity of mitochondrial respiratory chain enzymes. MPTP treatment significantly reduced the activity of Complex I and Complex II+III in mitochondrial respiratory chain. Interestingly, BDNF overexpression could restore the activity of these enzymes significantly (Figure 5a,b). We also examined the activity of Complex IV in the treated mice, and no difference was observed among different treated groups (Figure 5c). All these data suggested that BDNF overexpression could mitigate mitochondrial impairment and improve mitochondrial function in MPTP‐induced PD mouse model.

### 3.4 BDNF alleviates dopaminergic neuron loss in MPTP‐induced mice

**FIGURE 6:**
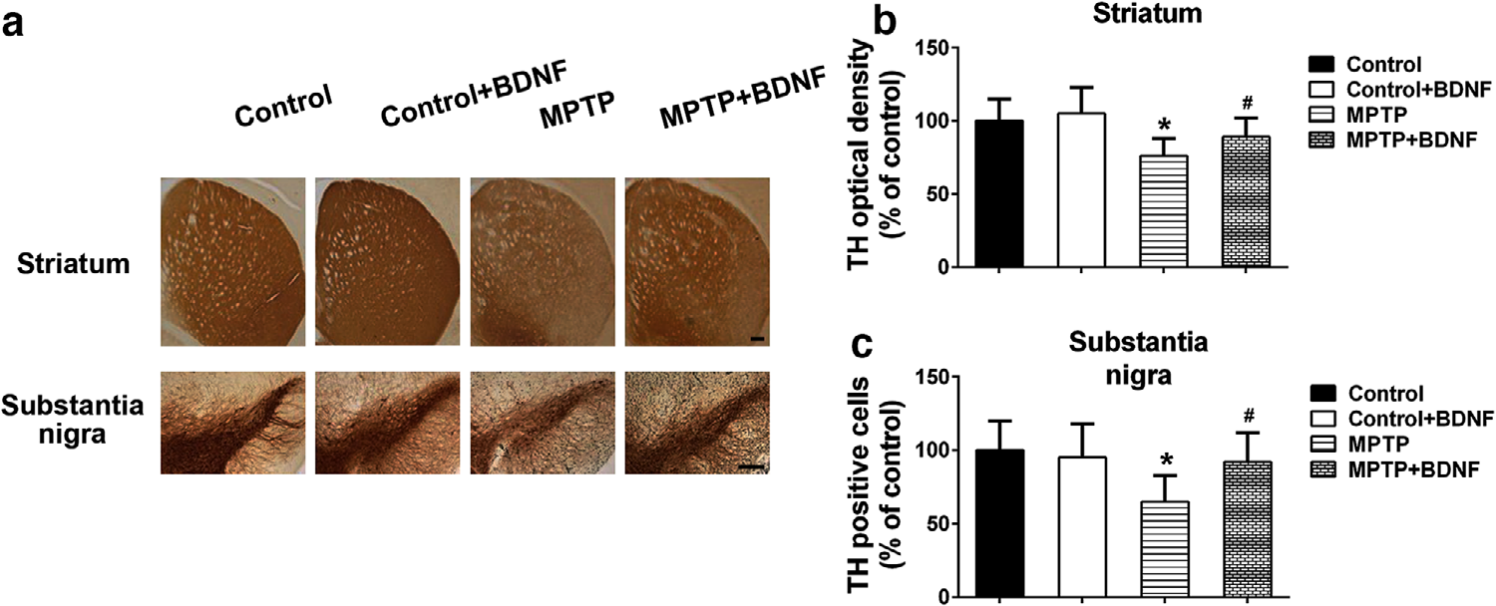
BDNF alleviated dopaminergic neuron loss in MPTP‐induced PD mice. (a) Brain sections were immunostained for TH immunoreactivity in striatum and substantia nigra. The TH optical density of striatum (b) and TH positive cells in substantia nigra (c) were quantified. Scale bars: 200 μm. Data were expressed as mean ± SD. *n* = 6 for each group. *P < .05 compared to control group, ^#^ *P*<.05 compared to MPTP group

Dopaminergic neuron loss in brain is another important feature of PD. Here, we evaluated the dopaminergic neuron status in different brain regions by detecting the immunoreactivity of TH. Immunostaining showed that MPTP treatment reduced the TH immunoreactivity in striatum (Figure 6a). Upon the overexpression of BDNF, TH immunoreactivity was significantly increased in striatum compared to that in MPTP alone treated mice (Figure 6a,b). Moreover, BDNF also restored TH immunoreactivity and alleviated dopaminergic neuron loss in substantia nigra in the MPTP‐induced PD mice (Figure 6a,c).

## 4 DISCUSSION

Mitochondria play critical roles in cellular signaling processes through regulating cellular energy metabolism to determine cell survival or death (Gorman et al., 2016). Therefore, mitochondria possess a critical role in the neurodegenerative diseases, such as AD, PD, and Huntington's disease (HD). PD is characterized by a series of motor and non‐motor disorders, such as tremor, rigidity, slowness of movement, cognitive dysfunctions, and memory, and affects about 1% of people over the age of 60 years (de Lau & Breteler, 2006). BDNF promotes neuro‐protection and neuro‐regeneration and presents as a promising agent in AD recently (Giuffrida et al., 2018). In this study, we delivered BDNF‐AAV into brain of MPTP‐induced PD mice and investigated the role of BDNF on motor and cognitive functions. BDNF overexpression significantly alleviated the MPTP‐induced impairments of on motor and cognitive functions though inhibiting the mitochondria damage of neurons in striatum and substantia nigra. Our finding suggested that BDNF overexpression had the neuron‐protective effect and could be a potential therapeutic strategy of PD treatment.

Motor and behavioral deficits are the major impairments for PD patients and some PD animal models. The mitochondrial toxin, MPTP, significantly induced motor deficits and cognitive impairments in mice. Rotarod test is one commonly used assay to evaluate balance, grip strength and motor coordination of mice (Carvalho et al., 2013), and pole test is widely used to assess basal ganglia related movement problems in mice (Doeppner et al., 2014). After 7 days of MPTP treatment, mice stayed short time on the rod (latency to fall) and spent long time to finish the pole test. However, BDNF overexpression could significantly attenuate these motor deficits (Figure 2). Moreover, BDNF was able to improve the cognitive impairments induced by MPTP in mice based on the open filed test and novel object recognition test, two commonly applied assays for exploratory activity, locomotor, learning, and memory ability (Antunes & Biala, 2012; Lopatina et al., 2014). The results of the above assays indicated that BDNF could effectively motor and non‐motor symptoms in mouse model.

BDNF is required for neuronal development, survival, and plasticity, and regulates the experience‐based synaptic strength and selective survival of neurons throughout the life (Licznerski & Jonas, 2018; Markham et al., 2004). In PD patients, the expression levels BDNF mRNA and protein are significantly reduced in the dopaminergic neurons of the substantia nigra (Chauhan et al., 2001; Howells et al., 2000). Porritt et al. (2005) reported that blocking the expression of BDNF could induce the loss of nigral dopaminergic neurons. Interestingly, mesencephalic culture experiments showed that BDNF treatment could promote sprouting of dopaminergic axons and improve the survival of dopaminergic neurons (Hyman et al., 1991). Therefore, decreased BDNF expression may contribute to the apoptosis of dopaminergic neurons in substantia and the progression of PD. In this study, BDNF overexpression decreased the expression of PINK1, an important protein that govern mitochondrial quality control (Pickrell & Youle, 2015), and restored the activity of key enzymes (Complex I, Complex Ⅱ+III) in mitochondrial respiratory chain. Markham et al. (2004) demonstrated that BDNF increased the efficiency of respiratory coupling of rat brain by activating MEK‐kinase of Complex I. BDNF showed dose‐dependent neuroprotective role in ibotenate injected mouse model via reduction of white matter cysts and grey matter lesions. In contrast, when rodent models were treated with BDNF antibody or MEK inhibitor, the neuroprotective effects of BDNF were blocked. Moreover, the BDNF‐induced positive effects on Complex I associated respiratory efficiency were suppressed (Gavillet et al., 2008; Hickman et al., 2008; Markham et al., 2012). The above findings suggested that BDNF might modify Complex I and generate concentration‐dependent promoting effects on ATP synthesis, respiratory coupling efficiency, and organelle integrity, and then enhance the respiratory control index finally. TH is the rate‐limiting enzyme in dopamine synthesis which is important for phenotypic expression (Daubner et al., 2011). MPTP treatment reduced TH immunoreactivity and decreased the number of TH positive neurons in striatum and substantia nigra, which is consistent with the dopaminergic neuron loss that parallels the PD's symptoms. Interestingly, the BDNF overexpression of brain can significantly improve MPTP‐induced dopaminergic neuron loss. In contract, blocking the expression of TH in SH‐SY5Y cells or striatum using rotenone, a mitochondrial inhibitor, could significantly induce the loss of TH positive dopaminergic neurons (Carriere et al., 2016; Ramalingam et al., 2019).

BDNF signaling regulates the survival, growth, differentiation, and plasticity of neurons through binding to tropomyosin receptor kinase B (TrkB) (Licznerski & Jonas, 2018). In the current study, BDNF present strong neuroprotective role on MPTP‐induced PD. However, overexpression BDNF in the brain of health mice did not show enhanced phenotypes on behavioral assessment and molecular activation, which might be due to the homeostasis regulating the downstream receptors or effectors (Reimers et al., 2014). It is necessary to identify the downstream effectors and dissect the underlying regulatory mechanism of BDNF mediated neuroprotective activity in PD and other neurodegenerative diseases.

## 5 CONCLUSION

This study demonstrated the neuroprotective effects and underlying mechanism of BDNF‐AAV on motor and non‐motor symptoms in MPTP‐induced PD mice, suggesting that delivery BDNF in brain through AAV is a potential therapeutic strategy for the treatment of mitochondrial impairment‐associated neurodegenerative diseases.

## CONFLICTS OF INTEREST

No competing financial interests exist.

### PEER REVIEW

The peer review history for this article is available at https://publons.com/publon/10.1002/brb3.2251.
